# Seroepidemiology of mumps in the general population of Jiangsu province, China after introduction of a one-dose measles-mumps-rubella vaccine

**DOI:** 10.1038/srep14660

**Published:** 2015-10-01

**Authors:** Yuanbao Liu, Ying Hu, Xiuying Deng, Zhiguo Wang, Peishan Lu, Fubao Ma, Minghao Zhou, Pei Liu, Jie Min

**Affiliations:** 1 Department of Expanded Programme on Immunization, Jiangsu Provincial Center for Disease Control and Prevention, Nanjing, Jiangsu province, 210009, China; 2 Department of Biostatistics and Epidemiology, School of Public Health, Southeast University, Nanjing, Jiangsu province, 210009, China

## Abstract

The mumps surveillance data from 2004 to 2011 showed that the incidence of mumps remained high after the one-dose measles-mumps-rubella (MMR) vaccine was introduced in China in 2008. A cross-sectional survey of mumps IgG in the general population of Jiangsu province was conducted in 2012 to gain comprehensive information on the immunity profile of the general population. The mean incidence was 15.2 per 100 000 individuals in Jiangsu province from 2004–2013. Two mumps incidence peaks were observed each year after introduction of the one-dose MMR vaccine. The seroprevalence did not significantly differ by region or sex, while the GMC significantly differed by region and sex. The overall GMC in Jiangsu province was 99.1 IU/ml (95% CI: 90.1–108.2), while the seroprevalence was only 59.1% (95% CI: 56.5–61.6). The seroprevalences for the 2 age groups that received the one-dose MMR vaccine, with reported coverage exceeding 95%, were 42.6% and 70.0%, respectively. The data on the incidence, MMR coverage, and seroprevalence in children younger than 6 years of age indicate that a two-dose MMR strategy should be considered. Mumps surveillance should be strengthened in children aged 6–11 and in those aged 12–17 because of their high contact rates and relatively low seroprevalences.

Mumps is a common childhood disease worldwide, especially in developing countries. It mainly causes benign infection in clinical settings, but some morbidities, such as orchitis, deafness, meningitis, and even death, have been consistently reported^1^. In China, 300–500 thousand cases were reported annually from 2004–2007 according to the Bulletin of the Ministry of Health^2^. In 2008, the measles-mumps-rubella virus (MMR) vaccine was introduced into the national immunization program (NIP), and it has since been routinely administered in a single dose to children aged 18–24 months. Similarly, in Jiangsu province, as an important part of the Expanded Program on Immunization (EPI), MMR vaccine administration was implemented on 1st May, 2008. Unfortunately, the mumps surveillance data from Jiangsu province indicates that the incidence of mumps declined for a short period from 2009–2010 and then increased again and remained high over the following years. In addition, while the reported MMR coverage in children aged 18 months to 6 years in Jiangsu province has exceeded 95% each year since 2009, the incidence of mumps in this age group has not declined since the introduction of the MMR vaccine. Further, the reported MMR coverage may not reflect the true coverage in the target population because of the large number of individuals in the floating population, especially the migrant workers and their children who came to the province due to the rapid economic development that has occurred in recent years. In addition, a certain proportion of floating children typically cannot be found through routine surveillance; therefore, vaccines cannot be administered to them, and they are definitely at high risk of mumps virus. Thus, a cross-sectional survey of the IgG antibody against the mumps virus in the general population of the Jiangsu province of China was carried out in 2012 to gain comprehensive information on the population’s immunity profile. Our findings may serve as a reference for the adjustment of the mumps vaccine strategy to control mumps in China in the future.

## Materials and Methods

### Mumps surveillance

Mumps is a class C infectious disease in China. All cases diagnosed by hospital staff or county-level centers for disease control and prevention are required to be recorded in the National Notifiable Disease Reporting System (NNDRS), a web-based computerized reporting system.

Mumps was confirmed in patients by laboratory testing, epidemiological linkage or clinical criteria, including acute onset of unilateral or bilateral, tender, self-limited swelling of the parotid or other salivary gland without another apparent cause. The case numbers were counted from the date of onset, incidence was calculated as the number of cases per 100 000 individuals, and the population denominators were provided by the National Bureau of Statistics of China.

### Serological survey

In 2012, a population-based, cross-sectional survey for IgG antibodies against the mumps virus was conducted in Jiangsu province. Jiangsu province is an eastern coastal province of China, with plains covering 68% and water covering another 18% of its total area. Most of Jiangsu has a humid subtropical climate, and it begins to transition into a humid continental climate in the north. The seasonal changes are clear-cut. The population density has reached 767/km^2^ according to the Sixth National Population Census. Based on the variations in geography, population density, climate, and socioeconomic status, the whole province was stratified into 2 regions (south and north). Two counties in the south region and 1 county in the north region were sampled at random. In each county, 3 villages or communities were randomly selected. In each village or community, information on all of the individuals was recorded and stratified by age. Then, a systematic sampling method was used for the selection and enrollment of subjects. Individuals within each selected county were sampled to be proportionally representative by age and gender.

A total of 1502 serum samples from individuals aged 1 month to 40 years were collected at the end of 2012. The samples from each region were stratified into the following 7 age groups: ≤18 months, 18–24 months, 3–5 years, 6–11 years, 12–17 years, 18–24 years, and 25–40 years. MMR immunization information on each child was retrospectively retrieved from the Jiangsu provincial vaccine and immunization system.

Approval for the study was obtained from the Medical Ethics Committee of the Jiangsu Provincial Center for Disease Control and Prevention. Written informed consent was provided by individuals or by the parents of children. The participants were asked to anonymously fill out a questionnaire that included personal information, such as sex, age or date of birth, and date of sampling. All methods were carried out in accordance with the approved guidelines.

### Laboratory assay

Serum samples were collected and stored at −70 °C before being tested. Serological tests were performed at the laboratory of the Department of Expanded Program on Immunization, Jiangsu Provincial Center for Disease Control and Prevention. Commercial ELISA kits (SERION ELISA classic anti-mumps virus IgG, InstitutVirion\Serion GmbH, batch number: SGB.CO) were used to detect and quantify human IgG antibodies against the mumps virus in sera. The control and standard sera were ready-to-use and did not require further dilution. For each test run, the control and standard sera were included independent of the number of microtiter test strips used, and the standard sera were tested in duplicate. In addition, in-house control samples were included with each assay run. To strictly control quality, we took the following precautions to avoid non-specific binding throughout the process: avoiding contamination and severe hemolysis during serum collection and separation; prohibiting repeated freeze-thawing of the serum; incubating the reagents at room temperature for 20 minutes before the start of the procedure; using aseptic techniques when removing aliquots from reagent tubes; and adequately washing and avoiding foaming^3^.

The ELISA results are expressed quantitatively as optical density (OD) measurements at 405 nm. The antibody concentration (IU/ml) was calculated using software from SERION and was then categorized as negative, equivocal, or positive using fixed cut-off values following international standards^4^,^5^,^6^. A value of greater than 108 IU/ml was considered positive, and a value of less than 108 IU/ml was considered negative. Samples with titers of between 90 IU/ml and 107 IU/ml were retested prior to categorization as positive or negative.

### Statistical analysis

Data from the questionnaires were double entered into Epidata software, with suitable edit checks and validations. The geometric mean concentrations (GMCs) and the antibody seroprevalence were calculated, in addition to their 95% confidence intervals (95% CIs), and the values were grouped by region, gender, and age.

Analysis of variance (ANOVA) was used to compare the GMCs, and a p-value of 0.05 was set as the significance threshold. The Student-Newman-Keuls q test (SNK-q test) was used for multiple comparisons. Differences among seroprevalences by region, sex, and age were assessed with Pearson’s χ^2^ test at a significance level of 0.05. R 2.10.0 statistical software was used for analyses.

## Results

### Changes in the epidemical characteristics of mumps from 2004 to 2013

A total of 116 289 cases were reported in Jiangsu province from 2004 to 2013 (the mean incidence was 15.2 per 100 000 individuals), and the maximum incidence was 24.3 per 100 000 individuals in 2013, while lowest incidence was 7.2 per 100 000 individuals in 2010. Although the incidence tended to decrease from 2009–2010 after introduction of the MMR vaccine in 2008, it rapidly increased after 2011 (see Table 1). The incidence of mumps varied by season, with the highest incidence from approximately April to July and a relatively high incidence from approximately October to December each year.

**Figure 1:**
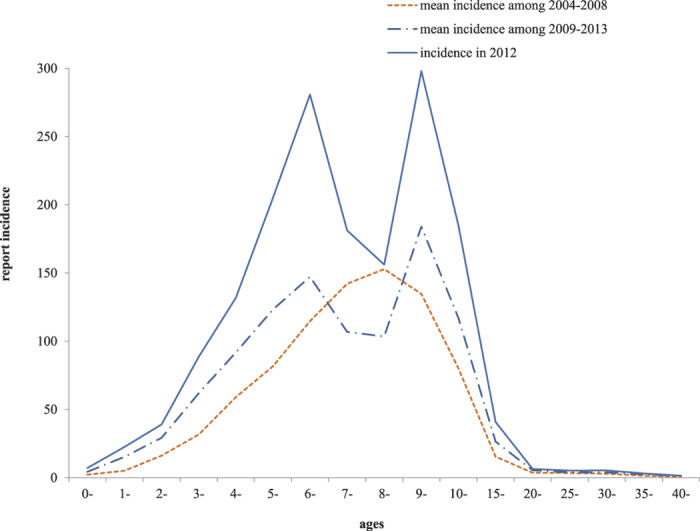
New epidemiological characteristics of mumps in Jiangsu province: correlation of mumps incidence with age before (2004–2008) and after (2009–2013) introduction of the one-dose MMR vaccine.

The mumps cases were distributed throughout the whole province. Furthermore, 50–75% of the cases were reported in the south region each year. Approximately 55–70% of the cases were kindergarten, primary school, junior high school, and senior high school students aged 3 to 18 years. From 2004–2008, prior to introduction of the one-dose MMR vaccine into the NIP, the incidence of mumps peaked each year in the children aged 8–9. After introduction of the one-dose MMR vaccine in 2009, the incidence exhibited two peaks each year, one for the children approximately 6 years of age, and the other for the children aged 10–15 years (see Fig. 1).

### Serosurvey of mumps antibodies in 2012

The seroprevalence did not significantly differ by region (north vs. south) or sex (male vs. female), while the GMC did significantly differ by region and sex. The GMC and seroprevalence were significantly lower in the children younger than 1.5 years of age compared with those in the other age groups. These two values were significantly lower in the children aged 1.5–2 years compared with those in the older age groups, and they tended to increase with age (see Table 2).

The vaccine effectiveness (VE) of one-dose MMR was calculated by combining the morbidity and vaccination data. The VE for the children aged 1.5–2 years and for those aged 3–5 years were 84.3% (95% CI: 30.5–96.5%) and 75.7% (95% CI: 38.0–90.4%), respectively (see Table 3).

**Figure 2:**
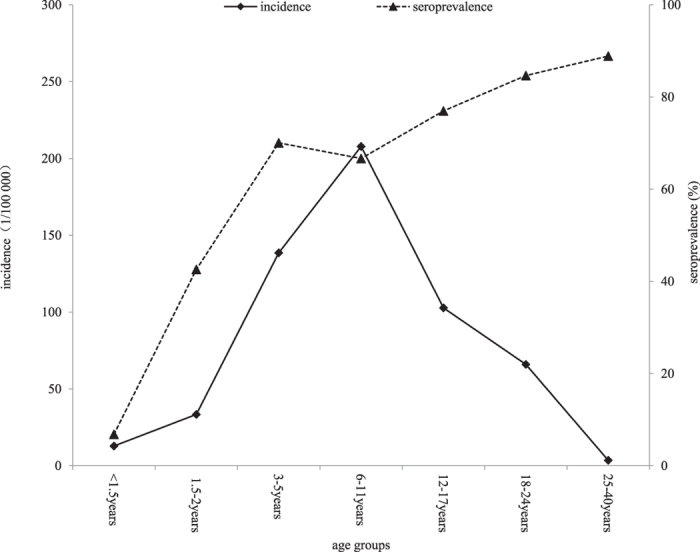
Relationship between incidence and seroprevalence by age.

The relationship between seroprevalence and incidence according to age is shown in Fig. 2. Among the children aged from 1.5 years to 11 years, the incidence tended to be positively correlated with the seroprevalence, and in those aged 12 to 40 years, the incidence tended to be negatively correlated with the seroprevalence.

**Figure 3:**
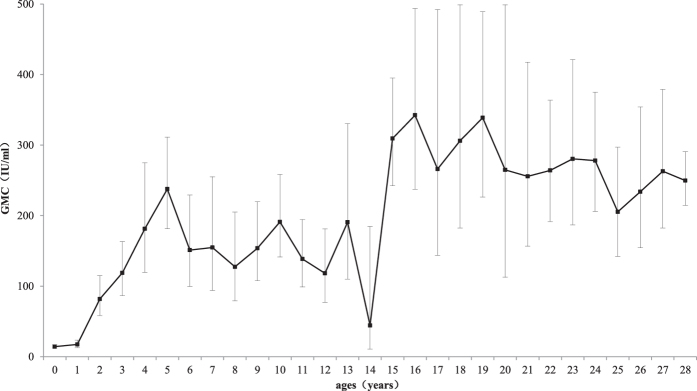
Age-specific geometric mean concentrations (GMCs) of mumps antibodies in Jiangsu province. The error bars represent 95% confidence intervals (CIs).

The age-specific GMC of mumps antibodies is shown in Fig. 3. The vaccination of children aged 1.5–2 years clearly induced a sharp increase in the level of mumps antibodies in the subsequent age cohorts up to age 5, with a GMC of 237.7 IU/ml (95% CI: 181.5–311.3 IU/ml). After age 5, this value declined to 44.3 IU/ml (95% CI: 10.6–184.6) by the age of 14 years. Thereafter, it remained relatively high at between 205.5 and 342.4 IU/ml.

**Figure 4:**
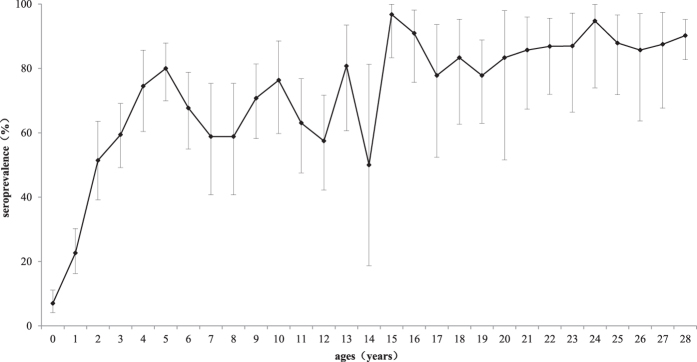
Age-specific seroprevalences of mumps antibodies in Jiangsu province. The error bars represent 95% confidence intervals (CIs).

The age-specific seroprevalence of mumps antibodies is shown in Fig. 4. After MMR vaccination at 18 months of age, the seroprevalence sharply increased to 80.0% (95% CI: 69.6–87.9%) in the children aged 5 years. Then, a fluctuation of between 50.0–80.7% was observed in those from 6 to 14 years of age. The seroprevalence remained high in the children in the older age groups.

## Discussion

Mumps is generally a childhood disease, and it is most common in children aged 5–9 years, while only a small proportion of adults are infected^1^,^7^,^8^. In countries where large-scale immunization against mumps has been implemented, the incidence of this disease has dramatically decreased. For example, in countries that have incorporated the one-dose mumps vaccine into their national immunization programs, the incidence of mumps has decreased by approximately 88–98%, while it has decreased by approximately 97–99% in countries that have implemented two-dose mumps vaccines^7^,^9^,^10^. However, our surveillance data showed that the incidence increased, and two peaks were observed for children aged 6 years and those aged 10–15 years after introduction of the one-dose MMR strategy in 2008. The epidemiological shift of disease incidence to the older age groups may potentially increase the rates of serious disease and complications^11^, although this situation has not been observed to deteriorate in Jiangsu province. Some studies have attributed this effect to the continuing low vaccination coverage^12^,^13^. In Jiangsu province, the reported coverage has exceeded 95% for the one-dose MMR vaccine in each cohort since 2008 because of the initiative to eliminate measles in China^3^,^14^. In addition, due to strict implementation of vaccination certificate inspection before the admission of children to kindergarten and primary school, at least 97% of children in kindergarten and primary school have received one dose of the MMR vaccine. However, the true coverage rate, including floating children throughout the entire province, remains unknown. Therefore, it may be necessary to conduct a coverage investigation in the target children aged 1.5–6 years, including the floating children, to clarify this issue.

With regard to the mumps incidence in children aged 7–8 years, as Fig. 2 shows, there was a remarkable decline, which may be attributed to the implementation of vaccination certificate inspection before the admission of children to primary school and the offering of one-dose MMR to unvaccinated children. However, it should be noted that the incidence in 7–8-year-old children was still relatively high.

The cross-sectional survey conducted in 2012 showed that the coverages of one-dose MMR vaccine in children aged 1.5–2 years and in those aged 3–5 years were 98.7% (153/155) and 97.0% (230/237), respectively. In contrast, the seroprevalences in children aged 1.5–2 years and in those aged 3–5 years were 42.6% (95% CI: 34.7–50.8%) and 70.0% (95% CI: 63.8–75.8%), respectively, which are far lower than the estimated herd immunity threshold of 86–92% for mumps^7^,^15^. Correspondingly, the incidences of mumps in children aged 1.5–2 years and in those aged 3–5 years (most of whom were in kindergarten) were 33.4 and 138.5 per 1000 000 individuals, respectively. Furthermore, mumps outbreaks frequently occurred in kindergartens each year.

These data indicate that the one-dose MMR vaccine has a limited protective effect on mumps virus in Jiangsu province, China. Multiple factors may have contributed to the lower seroprevalence after vaccination^9^,^16^. The mumps virus (MuV) has only one serotype but multiple genotypes. Studies of global mumps epidemics have defined 12 distinct MuV genotypes (A–L)^1^,^17^,^18^. Mumps epidemiological studies conducted in China have shown that the F genotype is the most common viral strain, whereas the predominant S79 vaccine contains the Jeryl Lynn strain with the A genotype^12^. The vaccine failure rate, i.e., the failure to induce antibodies to mumps caused by the S79 strain, may be relatively high for the one-dose MMR vaccine or monovalent mumps vaccine in China. Several mumps studies, especially the cohort studies conducted in China, have reported that the seroconversion rate of MMR is approximately 66–86%^19^,^20^, while that of the monovalent mumps vaccine is even lower, at 54%^21^. These rates are insufficient for mumps control^22^. Reports from other counties have indicated that the VE of the one-dose mumps vaccine containing the Jeryl Lynn strain is approximately 80%^1^,^23^,^24^. Similar results are depicted in Table 3, showing that the VE in children aged 1.5–2 years who have only received the one-dose MMR vaccine was 84.3% (95% CI: 30.5–96.5%). However, the VE in older children aged 3–5 years was decreased to 75.7% (95% CI: 38.0–90.4%). These findings may indicate that the protective effects of the one-dose MMR vaccine wane over time.

Theoretically, different vaccine strains can generate immune responses that recognize all genotypes. However, reports of mumps outbreaks in some countries in recent years have implied that limited antigenic cross-reactivity exists between different genotypes^10^,^25^,^26^. For example, the mumps virus outbreak in Jewish schools in America from 2009–2010 was due to genotype G. The nucleotide sequence of the SH gene revealed that the genetic distance between genotypes F and A was greater than that for other genotypes^12^,^13^. While the effects of strain variation on vaccine efficacy were not confirmed, cross-protection between the S79 strain, which belongs to the A genotype, and the F genotype may be lower^12^,^20^. Both the high incidence and low seroprevalence in children aged 1.5–2 years and in those aged 3–5 years indicate that the current vaccines cannot adequately protect children from genotype F. Furthermore, Table 2 shows that the GMC in children aged 3–5 years was higher than the positive threshold (108 IU/ml) but remained relatively low. Although the correlation between the GMC and protection from mumps has not yet been identified, evidence from other studies has shown that individuals with lower titers (GMC) to the mumps virus are more likely to show clinical signs of mumps after exposure^4^,^8^. Therefore, an increased level of age-specific immunity may be needed to protect school-aged children^23^,^27^.

In addition, high contact rates in kindergarten, primary school, and senior high school due to high population density may lead to mumps epidemics. Studies of mumps outbreaks in the United States have revealed that a high contact rate and population density can facilitate transmission (in settings such as colleges) and that such exposure may overcome vaccine-induced protection in students^7^,^26^,^28^. Similarly, most outbreaks that have occurred in kindergarten and primary schools in Jiangsu province may be attributed to high contact rates among the children and the lower seroconversion rate of the one-dose MMR vaccine.

Therefore, the one-dose MMR vaccine strategy may be insufficient, and two-dose vaccines should be considered. Many studies have reported that the effectiveness of the two-dose mumps vaccine in preventing clinical mumps is approximately 88%^24^,^29^,^30^. A serosurvey conducted on the Dutch population has suggested that the second MMR vaccination is crucial for maintaining a protective antibody level for a longer period of time and for maintaining the seroprevalence above the herd immunity threshold in the entire population^6^. Vandermeulen *et al.* have observed similar results, reporting that a two-dose MMR vaccine can provide significantly better humoral protection against mumps^31^.

Figure 2, which shows the relationship between seroprevalence and incidence in different age groups, also reveals interesting findings. Among the children aged 1.5 to 11 years, the incidence tended to be positively correlated with the seroprevalence, indicating that a seroprevalence of lower than 70% (see Table 2) would not prevent mumps epidemics. In the individuals aged 12 to 40 years, the incidence tended to be negatively correlated with the seroprevalence, indicating that a seroprevalence of higher than 70% may be essential for mumps control. When the seroprevalence was near the lower limit of the herd immunity threshold of 86%, such as the 84.7% value in the individuals aged 18–24 years, the mumps incidence dramatically declined.

Figures 3 and 4 show that vaccination of the children aged 1.5–2 years clearly induced a sharp increase in mumps antibodies and seroprevalence in the subsequent age cohorts until age 5. However, even the highest antibody titer observed in the 5-year-old children (237.7 IU/ml, 95% CI: 181.5–311.3 IU/ml) was still relatively low. Furthermore, the highest seroprevalence induced by the MMR vaccine in the 5-year-old children was only 80.0% (95% CI: 69.6–87.9%), which is lower than the herd immunity threshold. The seroprevalence was also low in the subsequent age group (6–14 years).

A higher GMC in the north than in the south is noted in Table 1, which may be attributed to the mumps epidemics that occurred in the north region from October 2011 to June 2012. Table 1 shows there were more mumps cases involving males than females, and Table 2 shows that the males had a significantly higher GMC than the females. These findings may be interpreted as indicating that the more intense exposure among the males compared with the females overwhelmed the protection afforded by the vaccine, resulting in a greater number of mumps infections^26^.

Our study was subject to some limitations. First, more than 90% of the mumps cases were clinically diagnosed over a surveillance period of ten years. However, we believe that the incidence of other types of parotitis was low because we adopted the strict clinical criteria mentioned above. Second, the immunization histories of nearly all of the children older than 6 years of age were unknown in the serosurvey.

In conclusion, both the GMC and seroprevalence in the children under 6 years of age were low and were far less than the herd immunity threshold. Furthermore, the one-dose MMR vaccine strategy may have a limited effect on mumps control, and a two-dose strategy should be considered. Mumps surveillance should be strengthened in children aged 6–11 years and in those aged 12–17 years because of their high contact rates and relatively low seroprevalences.

## Additional Information

**How to cite this article**: Liu, Y. *et al.* Seroepidemiology of mumps in the general population of Jiangsu province, China after introduction of a one-dose measles-mumps-rubella vaccine. *Sci. Rep.* **5**, 14660; doi: 10.1038/srep14660 (2015).

## Figures and Tables

**Table 1:** Reported cases and incidence among 2004–2013 in Jiangsu province, China.

Year	Incidence (1/100000)	Cases (N,%)				
		N	clinically diagnosed	laboratory confirmed	male	female
2004	13.3	10028	9606(95.8)	422(4.2)	6393(63.8)	3635(36.2)
2005	12.2	9111	9034(99.2)	77(0.8)	5884(64.6)	3227(35.4)
2006	12.5	9326	9277(99.5)	49(0.5)	6076(65.2)	3250(34.8)
2007	14.3	10824	10753(99.3)	71(0.7)	7125(65.8)	3699(34.2)
2008	18.4	14004	13919(99.4)	85(0.6)	8928(63.8)	5076(36.2)
2009	13.2	10134	10093(99.6)	41(0.4)	6449(63.6)	3685(36.4)
2010	7.2	5557	5518(99.3)	39(0.7)	3569(64.2)	1988(35.8)
2011	11.3	8893	8855(99.6)	38(0.4)	5696(64.1)	3197(35.9)
2012	24.2	19131	19086(99.8)	45(0.2)	12469(65.2)	6662(34.8)
2013	24.3	19281	19256(99.9)	25(0.1)	12245(63.5)	7036(36.5)

**Table 2:** GMCs and seroprevalence of mumps antibody by region, sex and age in Jiangsu province, China, in 2012 (IU/ml).

Characteristics		sample size	GMC	95% CI	seroprevalence	95% CI
region	north	500	117.6^a^	(101.6,136.1)	63.2	(58.8,67.4)
	south	1002	90.9	(81.3,101.5)	57.0	(53.9,60.1)
sex	male	792	111.9b	(98.8,126.6)	62.0	(58.5,65.4)
	female	710	86.4	(76.1,98.1)	55.8	(52.0,59.5)
age	<1.5 years	294	12.6^c^	(10.8,14.6)	6.8	(4.2,10.3)^c^
	1.5–2 years	155	48.7^c^	(36.7,64.6)	42.6	(34.7,50.8)^c^
	3–5 years	237	166.9	(138.0,201.9)	70.0	(63.8,75.8)
	6–11 years	282	151.7	(129.0,178.3)	66.7	(60.8,72.1)
	12–17 years	165	194.5	(157.7,239.9)	77.0	(69.8,83.2)
	18–24 years	189	287.8	(244.7,338.5)	84.7	(78.7,89.5)
	25–40 years	180	240.8	(212.5,272.9)	88.9	(83.4,93.1)
total		1502	99.1	(90.1,108.2)	59.1	(56.5,61.6)

Abbreviations: CI, confidence interval; GMC, geometric mean concentrations

significantly lower than other age groups, p < 0.05.

^a^north vs. south, p < 0.05.

^b^male vs. female, p < 0.05.

**Table 3:** Vaccine effectiveness (VE) of one dose MMR.

age group	MMR status	N	mumps cases(N)	VE(%,95% CI)
1.5–2 years	MMR vaccinatied	153	12	84.3(30.5,96.5)
	MMR unvaccinatied	2	1	
3–5 years	MMR vaccinatied	230	24	75.7(38.0,90.4)
	MMR unvaccinatied	7	3	
